# Physiology, monitoring, and optimisation of perioperative tissue oxygenation: a narrative review

**DOI:** 10.1016/j.bja.2026.01.047

**Published:** 2026-04-13

**Authors:** Jens Meier, Sigismond Lasocki, Patrick Meybohm, Daniela Filipescu, Thorsten Haas, Julien Pottecher, Emmanuel Rineau, Stefano Romagnoli, Alina Bergholz, Bernd Saugel

**Affiliations:** 1 Department of Anesthesiology and Critical Care Medicine, Kepler University, Hospital GmbH and Johannes Kepler University, Linz, Austria; 2 Department of Anesthesia and Critical Care, University Hospital of Angers, Angers, France; 3 University Hospital Würzburg, Department of Anaesthesiology, Intensive Care, Emergency and Pain Medicine, Würzburg, Germany; 4 Department of Cardiac Anaesthesia and Intensive Care, Emergency Institute for Cardiovascular Diseases, University of Medicine and Pharmacy Carol Davila, Bucharest, Romania; 5 Department of Anesthesiology, University of Florida Health, Gainesville, FL, USA; 6 Department of Anesthesiology, Critical Care & Perioperative Medicine, FHU DATA-SURGE, FMTS, Strasbourg University Hospital, Strasbourg, France; 7 Department of Health Science, University of Florence, Florence, Italy; 8 Department of Anesthesia and Intensive Care, Azienda Ospedaliero-Universitaria Careggi, Florence, Italy; 9 Department of Anesthesiology, Center of Anesthesiology and Intensive Care Medicine, University Medical Center Hamburg-Eppendorf, Hamburg, Germany; 10 Outcomes Research Consortium®, Houston, TX, USA

**Keywords:** arterial pressure, cardiac output, haemodynamic monitoring, microcirculation, mitochondria, oxygen delivery, tissue perfusion

## Abstract

Maintenance of tissue oxygenation in patients having surgery is important as tissue hypoxia is a major determinant of organ failure. Tissue oxygenation follows a stepwise physiological pathway involving the macrocirculation, the microcirculation, and the cellular oxygen metabolism. This narrative review endorsed by the Network for the Advancement of Patient Blood Management, Haemostasis and Thrombosis outlines the physiology of tissue oxygenation, evaluates methods for intraoperative tissue oxygenation monitoring, and summarises therapeutic strategies to ensure adequate tissue oxygenation. In the macrocirculation, oxygen is delivered to peripheral organs by convection (through the bulk flow of oxygenated blood generated by cardiac output). Effective tissue perfusion requires both sufficient blood flow and perfusion pressure. Interventions targeting the macrocirculation include fluid therapy, blood transfusions, and targeted management of arterial pressure and cardiac output. Within the microcirculation, oxygen diffuses from capillaries into the surrounding tissues. The microcirculation distributes blood flow according to local metabolic demands. Most techniques for intraoperative microcirculation monitoring, such as handheld vital microscopy, near-infrared spectroscopy, laser Doppler, laser speckle imaging, fluorescence angiography, or the urethral perfusion index, are not implemented in clinical practice. The role of therapeutic interventions specifically targeting the microcirculation remains uncertain. At the cellular level, oxygen is consumed within the mitochondria, where it serves as the final electron acceptor in oxidative phosphorylation to generate adenosine triphosphate. Direct monitoring of cellular oxygen metabolism remains experimental and is not routinely available. Therapeutic strategies aiming to directly improve cellular oxygen metabolism are evolving. Future research is needed to better understand how to optimise tissue oxygenation during surgery to improve patient-centred outcomes.

**Editor’s key points**

• Adequate tissue oxygenation is essential for maintaining organ function during and after surgery.
• Most clinical strategies for intraoperative haemodynamic management focus on the macrocirculation, but tissue oxygenation also depends on the microcirculation and cellular oxygen metabolism, which are more challenging to measure clinically.
• Monitoring and treatment of tissue oxygenation should address the complete pathway of oxygen delivery from macrocirculation to microcirculation to mitochondrial uptake and reduction.
• Future research is needed to develop improved monitors and treatments of microcirculatory function, oxygen delivery and metabolism.

Tissue hypoxia is a major determinant of organ failure in patients having surgery.^1^^,^^2^ Therefore, ensuring adequate tissue oxygenation to prevent tissue oxygen debt must guide perioperative treatment decisions, including haemodynamic and fluid management, ventilation, and blood transfusion. However, the physiology and pathophysiology of tissue oxygenation are complex, involving a delicate interaction between the macrocirculation, the microcirculation, and cellular oxygen metabolism. With routine intraoperative monitoring,^3^^,^^4^ many components of tissue oxygenation remain unmeasured.

Advanced monitoring approaches could potentially help identify and understand alterations in tissue oxygenation. In this narrative review, we consider the physiology of tissue oxygenation, integrating the macrocirculation, the microcirculation, and cellular oxygen metabolism; evaluate the methods available for intraoperative monitoring of tissue oxygenation at every level of the oxygen cascade; and summarise the evidence on therapeutic strategies to ensure adequate tissue oxygenation to eventually improve patient-centred outcomes.

## Physiology of tissue oxygenation: macrocirculation, microcirculation, and cellular oxygen metabolism

**Fig 1:**
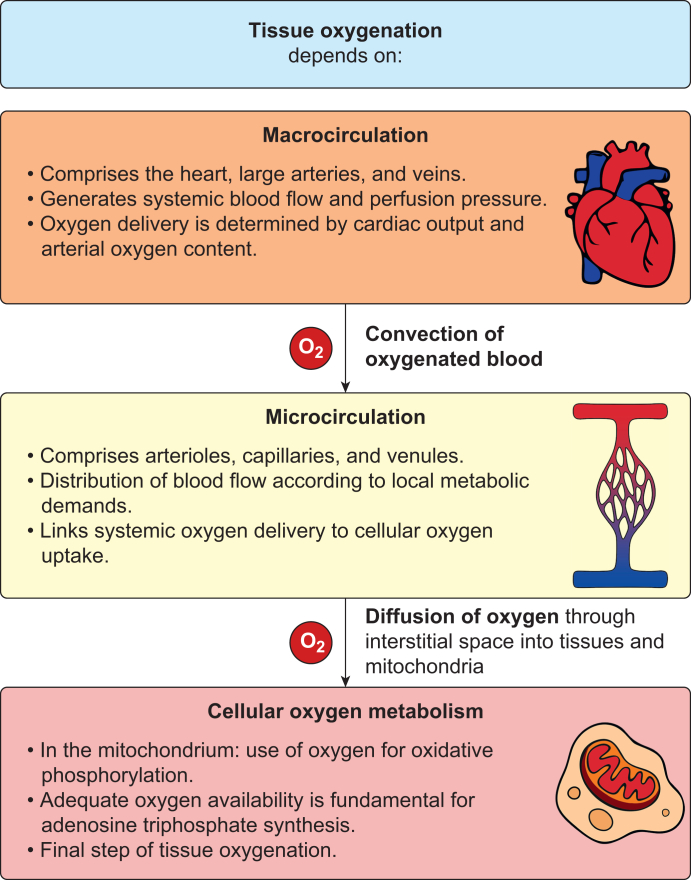
Physiological pathway of tissue oxygenation. Tissue oxygenation depends on a physiological pathway involving the macrocirculation, the microcirculation, and cellular oxygen metabolism.

Oxygen transport to the tissues follows a stepwise physiological pathway from the macrocirculation to the microcirculation to cellular oxygen metabolism (Fig. 1). The macrocirculation delivers oxygen to peripheral organs by convection through the bulk flow of oxygenated blood generated by cardiac output.^3^^,^^4^ Within the microcirculation, oxygen diffuses from capillaries into the surrounding tissues.^5^, ^6^, ^7^ At the cellular level, oxygen is consumed within mitochondria, where it serves as the final electron acceptor in oxidative phosphorylation to generate adenosine triphosphate (ATP).^8^^,^^9^ Haemoglobin plays a central role as the primary carrier of oxygen in the blood. By binding oxygen molecules in the lungs and releasing them in peripheral tissues, haemoglobin is critical for oxygen transport and delivery, both systemically and at the cellular level.

### Macrocirculation

**Fig 2:**
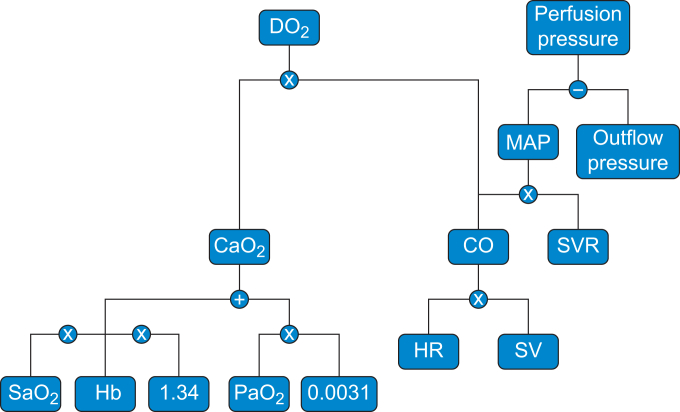
Determinants of oxygen delivery and perfusion pressure. Oxygen delivery (DO_2_) is the product of cardiac output (CO) and arterial oxygen content (CaO_2_). CO is determined by heart rate (HR) and stroke volume (SV). CaO_2_ depends on haemoglobin concentration (Hb), arterial haemoglobin oxygen saturation (SaO_2_), and the arterial partial pressure of oxygen (PaO_2_), using the constants 1.34 ml O_2_ per g Hb and 0.0031 as the solubility coefficient of oxygen in plasma. Perfusion pressure is calculated as mean arterial pressure (MAP) minus outflow pressure (e.g. central venous or compartment pressure). MAP results from CO and systemic vascular resistance (SVR).

The macrocirculation comprises the heart, large arteries, and veins and generates systemic blood flow and perfusion pressure (Fig. 2).^3^ The physiological variable summarising the contribution of macrocirculation to tissue oxygenation is oxygen delivery, which quantifies the total amount of oxygen transported from the lungs to the tissues per minute. It is the product of cardiac output (the volume of blood ejected by the heart per minute) and arterial oxygen content (the amount of oxygen carried in arterial blood):

OxygenDelivery=ArterialOxygenContent×CardiacOutput

The arterial oxygen content depends on both the amount of oxygen bound to haemoglobin and the amount of oxygen dissolved in plasma. The amount of oxygen bound to haemoglobin, in turn, depends on the haemoglobin concentration and the arterial oxygen saturation (i.e. the proportion of haemoglobin carrying oxygen). The amount of oxygen dissolved in plasma depends on the arterial partial pressure of oxygen. The relationship is described by the following equation:

$$\text{Arterial}\;\text{Oxygen}\;\text{Content}=\left(\text{Arterial}\;\text{Oxygen}\;\text{Saturation}\times \text{Haemoglobin}\times 1.34\right)+\left(\text{Arterial}\;\text{Partial}\;\text{Pressure}\;\text{of}\;\text{Oxygen}\times 0.0031\right)$$

The constant 1.34 corresponds to the amount of oxygen in ml that can bind to 1 g of haemoglobin, and 0.0031 is the solubility coefficient of oxygen in plasma at 37°C. In most physiological states, oxygen bound to haemoglobin accounts for most of the arterial oxygen content, while the dissolved oxygen fraction contributes only minimally.

Effective tissue perfusion requires sufficient blood flow, which depends on perfusion pressure. Global blood flow can be quantified by cardiac output, the product of left ventricular stroke volume and heart rate. Stroke volume depends on ventricular preload, myocardial contractility, and afterload, and therefore reflects the heart’s ability to generate forward flow under varying conditions. The interaction of stroke volume with the mechanical properties of the arterial tree determines pulse pressure (the difference between systolic and diastolic arterial pressure). Heart rate can be affected by various factors in patients having surgery with general anaesthesia. There is no evidence-based definition for ‘intraoperative bradycardia’ or ‘intraoperative tachycardia.’ High intraoperative heart rates can occur as a consequence of intravascular hypovolaemia, inadequate anaesthesia, or systemic inflammation and can increase myocardial oxygen consumption and decrease myocardial oxygen supply. Low heart rates can result from anaesthetic or vasoactive medications or parasympathetic system-mediated reflexes and can result in low cardiac output and hypotension.

The primary determinant of perfusion pressure is mean arterial pressure, which is the inflow pressure for most organs.^10^^,^^11^ Mean arterial pressure represents the average pressure during one cardiac cycle. Perfusion pressure is the gradient between inflow pressure (usually mean arterial pressure) and outflow pressure. The outflow pressure differs between organs, reflecting anatomical characteristics and venous drainage pathways. Under physiologic conditions, the outflow pressure is determined by central venous pressure. However, in some organs or compartments, regional tissue pressures can exceed central venous pressure and thereby become the dominant regional outflow pressure. For example, elevated intracranial pressure can exceed central venous pressure and thus determine cerebral venous outflow; elevated intra-abdominal pressure can limit renal, hepatic, and splanchnic venous drainage; elevated intramuscular or compartment pressures can impair venous outflow from the limbs; elevated intrathoracic pressure (e.g. during mechanical ventilation) can affect pulmonary venous return; and elevated pericardial pressure, as in pericardial effusion, can restrict cardiac venous drainage. Venous excess ultrasound (VExUS) can help assess venous congestion and estimate outflow pressure in the liver and kidney,^12^ although it is difficult to apply intraoperatively. Inadequate perfusion pressures, whether because of low mean arterial pressure or high central venous or compartmental pressure, can compromise regional tissue perfusion and oxygen delivery, even when global oxygen delivery appears adequate.

Mean arterial pressure is incorrectly regarded as a surrogate for cardiac output in perioperative care by assuming a stable relationship between mean arterial pressure, cardiac output, and systemic vascular resistance. However, the heart and the arterial system are anatomically and functionally linked structures, a concept described as ‘ventriculo-arterial coupling.’^13^^,^^14^ Ventriculo-arterial coupling describes the relationship between the pumping ventricle and the effective load of the arterial system against which the heart pumps, and it reflects how efficiently the heart ejects blood into the vascular system.^13^^,^^14^ Ventriculo-arterial coupling is quantified as the ratio of arterial elastance to ventricular elastance and allows assessing cardiovascular performance by integrating both ventricular and arterial function.^13^^,^^14^ Changes in myocardial contractility, arterial tone, or arterial compliance alter this relationship, making mean arterial pressure an unreliable indicator of cardiac output during general anaesthesia and surgery.^15^ Relying solely on mean arterial pressure thus may obscure relevant haemodynamic alterations.^4^

By establishing both the convective flow of oxygenated blood and the perfusion pressure, the macrocirculation serves as the upstream driver of oxygen transport to the microcirculation and ultimately to the mitochondria.

### Microcirculation

The microcirculation comprises arterioles, capillaries, and venules with luminal diameters of 5–100 μm.^16^ It provides the transition from high-volume convective flow of oxygenated blood to diffusion of oxygen from the capillaries through the interstitial space into the surrounding tissues and mitochondria, thereby linking systemic oxygen delivery to cellular oxygen uptake.^5^, ^6^, ^7^ The primary physiological roles of the microcirculation are to distribute blood flow according to local metabolic demands by modulating vascular tone through endogenous vasomotor activity and to maintain vascular barrier function through an intact endothelial lining and protective glycocalyx.

**Fig 3:**
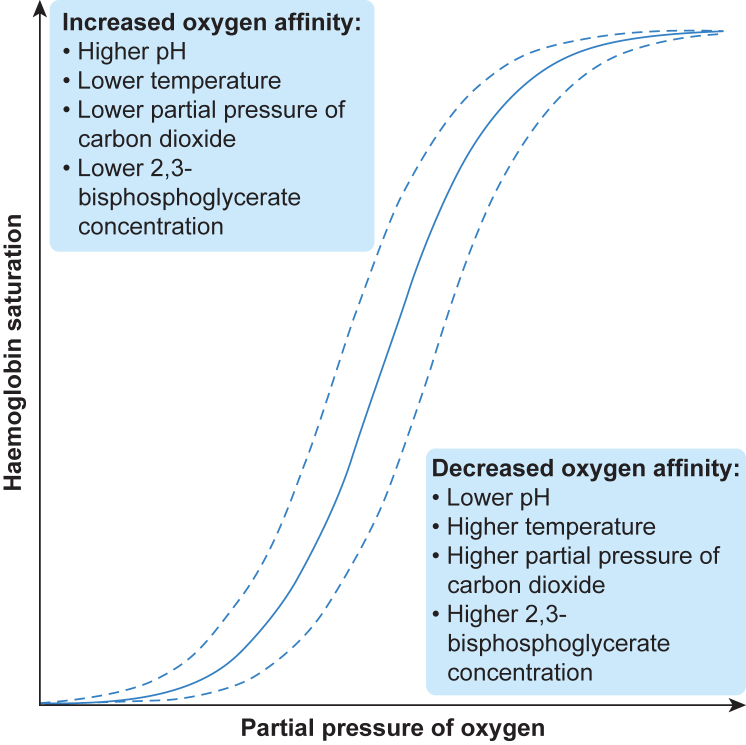
Oxygen-haemoglobin dissociation curve. Relationship between partial pressure of oxygen and haemoglobin saturation, including factors that shift haemoglobin-oxygen affinity.

Within the microcirculation, haemoglobin both carries oxygen and ensures coupled regulation of oxygen release according to local metabolic factors. Changes in pH, temperature, partial pressure of carbon dioxide, and 2,3-bisphosphoglycerate concentration modulate the affinity of haemoglobin for oxygen, allowing haemoglobin to release oxygen in areas of increased metabolic activity (Fig. 3).^17^

The rheological properties of blood also influence microcirculatory blood flow. Blood viscosity, which is determined by shear rate, plasma viscosity, and haematocrit, decreases with increasing shear rates, a phenomenon known as shear thinning.^18^ The deformability of erythrocytes is essential for maintaining blood flow in high-shear conditions, whereas the aggregability of erythrocytes influences blood flow in low-shear conditions. Increased interactions between leucocytes and the endothelium also increase flow resistance, particularly in low-flow states.^19^ Elevated haematocrit exacerbates these interactions. Erythrocytes further modulate local vasodilation and thus influence microvascular perfusion through haemoglobin-mediated nitric oxide scavenging, and under certain conditions, nitric oxide release from S-nitrosohaemoglobin.^20^ Finally, the dense and complex architecture of the microvascular network itself contributes to microvasculature flow resistance.^21^

In the tissues, oxygen diffuses through the interstitial space and across the cell membrane to mitochondria down a concentration gradient from the higher partial pressure in blood to the lower partial pressure in cells.^5^, ^6^, ^7^ This process depends on both the diffusion distance and the properties of the surrounding tissue, particularly capillary vessel density and membrane architecture.

**Fig 4:**
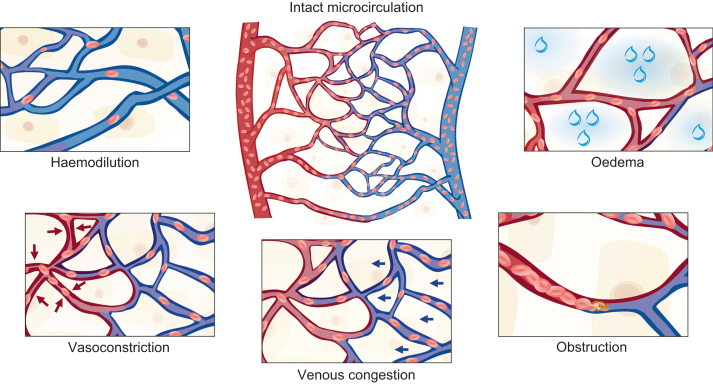
Mechanisms of impaired microcirculation. The microcirculation can be impaired by vasoconstriction, obstruction, venous congestion, haemodilution, or interstitial oedema. These mechanisms can reduce microcirculatory blood flow or capillary vessel density, limiting oxygen delivery at the microcirculatory level. Reprinted from: Flick and colleagues ^16^. © 2024 with permission from Elsevier.

The microcirculation can be impaired during vasoconstriction (e.g. in shock states or with high-dose vasopressor therapy), obstruction (e.g. microthrombosis in sepsis), venous congestion (e.g. fluid overload or elevated central venous pressure), haemodilution (low haematocrit, thereby reducing oxygen-carrying capacity), or interstitial oedema (increased tissue pressure impairing capillary flow and increased oxygen diffusion distance), as illustrated in Fig. 4.^16^ Although macrocirculatory variables such as mean arterial pressure and cardiac output influence the microcirculation, an apparently normal macrocirculation does not ensure adequate microcirculatory perfusion. Patients with severe sepsis, major trauma, severe acute pancreatitis, or major burns can have profound microcirculatory dysfunction despite normal or even high macrocirculatory variables.^22^, ^23^, ^24^, ^25^ This phenomenon, referred to as ‘loss of haemodynamic coherence’ or ‘haemodynamic decoupling’,^26^, ^27^, ^28^ reflects a disconnection between the macrocirculation and the microcirculation. This disconnection might help explain why haemodynamic treatment strategies focusing on macrocirculatory optimisation alone often fail to improve outcomes in severe inflammatory conditions.^29^

### Cellular oxygen metabolism

At the cellular level, oxygen serves as the final electron acceptor in mitochondrial oxidative phosphorylation, the process by which most ATP is generated to support essential cellular functions.^8^^,^^9^^,^^30^ During the oxidation of carbohydrates, fatty acids, and amino acids, electrons are transferred to redox coenzymes. These coenzymes donate electrons to the mitochondrial respiratory chain, where a proton gradient is established across the inner mitochondrial membrane. This electrochemical gradient drives the synthesis of ATP via ATP synthase, with molecular oxygen being reduced to water in the final step.^30^

Oxidative phosphorylation is highly efficient.^30^ In contrast, when oxygen availability is insufficient, cells must rely on anaerobic glycolysis, in which glucose is metabolised to lactate in the cytosol. This pathway is far less efficient than oxidative phosphorylation, generating about 90% less ATP per glucose molecule.^31^ As a result, inadequate oxygen supply compromises ATP production, impairs protein synthesis and cellular repair, and compromises cell integrity. Adequate oxygen availability is therefore fundamental to maintaining mitochondrial ATP production and preserving cellular homeostasis.

Effective oxygen use at the cellular level depends not only on oxygen supply but also on mitochondrial integrity, enzymatic function, and the redox state of the cell.^32^^,^^33^ The erythrocyte actively contributes to cellular oxygen metabolism by producing nicotinamide adenine dinucleotide phosphate via the pentose phosphate pathway, which protects it from oxidative stress and preserves its capacity for effective oxygen transport.^34^ These auxiliary roles close the loop between convective blood flow and systemic oxygen delivery, microcirculatory distribution, and cellular oxygen metabolism. Thus, changes in haemoglobin concentration or erythrocyte function can disrupt the entire pathway from oxygen transport to oxygen consumption.

## Methods for intraoperative tissue oxygenation monitoring

To ensure adequate tissue oxygenation in patients having surgery, the various determinants of tissue oxygenation must be measured. While monitoring of basic macrohaemodynamic variables is mandatory during surgery,^4^ and advanced macrohaemodynamic monitoring is also widely available,^3^ direct monitoring of the microcirculation and cellular oxygen metabolism is not routinely performed and remains mainly experimental.

### Macrocirculation

Obligatory basic intraoperative monitoring includes electrocardiography, arterial pressure monitoring, and pulse oximetry.^4^ These monitors provide basic information on perfusion pressure but offer only indirect and limited insights into oxygen delivery and tissue oxygenation. Advanced macrocirculatory monitoring can provide a more comprehensive assessment of key determinants of perfusion pressure and oxygen delivery.^3^ Perfusion pressure and oxygen delivery monitoring each require distinct monitoring methods.

Monitoring of perfusion pressure requires measurements of inflow pressure (usually mean arterial pressure^10^^,^^11^) and outflow pressure (central venous pressure or compartment pressure). Mean arterial pressure can be measured noninvasively and intermittently using oscillometry, invasively and continuously using arterial catheterisation, or noninvasively and continuously using newer methods, such as finger-cuff systems.^35^, ^36^, ^37^, ^38^, ^39^ Continuous, compared to intermittent, arterial pressure monitoring can help clinicians detect and mitigate hypotension during induction of anaesthesia and during surgery.^40^, ^41^, ^42^, ^43^

Central venous pressure is measured through a central venous catheter inserted into the superior vena cava.^44^ Regional tissue pressures can be measured using compartment-specific methods, for example, intra-abdominal pressure through bladder pressure measurements^45^ or intracranial pressure through intraparenchymal probes or external ventricular drains.^46^ However, central venous catheters are reserved for high-risk patients or high-risk surgery, and regional tissue or compartment pressures are not routinely measured. Consequently, in clinical practice, mean arterial pressure is often the only pressure monitored during surgery, and perfusion pressure is typically approximated by inflow pressure alone, a simplification that overlooks venous or compartmental pressures and can overestimate actual perfusion pressure. It has thus been recommended that outflow pressure should be considered when defining mean arterial pressure targets.^47^

Monitoring of oxygen delivery requires measurements of cardiac output, haemoglobin concentration, arterial oxygen saturation, and arterial partial pressure of oxygen. However, even in high-risk patients, stroke volume and cardiac output are rarely measured during noncardiac surgery.^48^ Cardiac output can be measured using invasive reference methods, such as pulmonary artery and transpulmonary thermodilution, or using minimally invasive or noninvasive methods, including pulse wave analysis, oesophageal Doppler ultrasonography, or bioreactance/bioimpedance.^49^, ^50^, ^51^ Currently, pulse wave analysis, the mathematical analysis of the arterial pressure waveform,^52^, ^53^, ^54^ is the most frequently used method to measure cardiac output in noncardiac surgery patients.^48^ Haemoglobin concentration, arterial oxygen saturation, and arterial partial pressure of oxygen can be intermittently measured using arterial blood gas analysis, offering a snapshot of arterial oxygen content.

### Microcirculation

Even when macrocirculatory variables appear normal, tissue perfusion can be critically impaired.^26^, ^27^, ^28^ Therefore, it would be important to monitor the microcirculation. Although several techniques for intraoperative monitoring of the microcirculation have been proposed,^16^ most of them are still experimental.

To visualise the microcirculation directly, handheld vital microscopy is a promising research tool that captures live images of the microvascular network for subsequent offline analysis.^55^ Handheld vital microscopy can display erythrocytes in small vessels such as arterioles, capillaries, and venules at the surface of accessible tissues.^55^ Video sequences can be analysed for total vessel density, proportion of perfused vessels, and microvascular flow index, each reflecting distinct aspects of microvascular function.^55^ In clinical studies, measurements are most commonly performed sublingually because the tongue is richly vascularised and easily accessible. The sublingual microcirculation is often used as a surrogate for systemic microvascular function. However, microcirculatory alterations vary among organ systems under pathophysiological conditions, and the sublingual microcirculation might not reflect regional microcirculation in other organs. The integration of handheld vital microscopy for routine real-time intraoperative monitoring is currently hindered by technical challenges, including requirements for specialised operator training and time-consuming offline video analysis.^56^^,^^57^

Near-infrared spectroscopy is a noninvasive monitoring technique that provides information about regional tissue oxygen saturation.^58^^,^^59^ By measuring the relative absorption of light by oxygenated and deoxygenated haemoglobin, near-infrared spectroscopy reflects the balance between oxygen delivery and consumption in small vessels, predominantly determined by the venous component of the microcirculation. Probes are commonly applied to the forehead. Low cerebral near-infrared spectroscopy values can be associated with postoperative complications, including delirium,^60^^,^^61^ but the clinical use of near-infrared spectroscopy (specifically its potential impact on patient-centred outcomes) remains a subject of ongoing and controversial research.^58^^,^^59^ There is considerable inter-individual variability in absolute values of regional oxygen saturation. Therefore, dynamic near-infrared spectroscopy tests, such as the vascular occlusion test, can yield more detailed information. In this test, a probe is typically placed over the thenar muscle, and the desaturation slope after inflation of a cuff around the forearm reflects local oxygen consumption, while the re-saturation slope after cuff release provides insights into microvascular reserve and endothelial function.^22^^,^^62^, ^63^, ^64^ However, interpretation of near-infrared spectroscopy values is complicated by several factors. Changes in skin blood flow can affect the signal, the monitoring depth of near-infrared spectroscopy varies across devices and patients, and the relative contributions of arterial, capillary, and venous blood are not constant.^65^ Baseline values therefore differ widely, and universal thresholds have not been defined.^66^

In addition to handheld vital microscopy and near-infrared spectroscopy, other techniques have been explored to assess microcirculatory perfusion during surgery. Optical methods such as laser Doppler ultrasonography^67^ and laser speckle imaging^68^ can provide perfusion maps by detecting the movement of erythrocytes in superficial vessels of the skin or exposed organ surfaces. These measurement techniques are highly motion-sensitive and have rarely been studied in intraoperative contexts. Fluorescence angiography, using intravenous dyes such as indocyanine green, offers a dynamic visualisation of tissue perfusion and has gained popularity in surgical disciplines including colorectal and plastic surgery.^69^, ^70^, ^71^ Although it provides valuable anatomical information, its interpretation remains largely qualitative and depends on dye dose, timing, and camera settings.

Microcirculatory perfusion is also reflected in the peripheral perfusion index derived from pulse oximetry,^72^ the urethral perfusion index,^73^ and peripheral temperature gradients. Although these variables are easily obtained and require minimal equipment, prospective studies showing their value for guiding intraoperative therapy or improving patient-centred outcomes are lacking.

### Cellular oxygen metabolism

Direct monitoring of cellular oxygen metabolism would be an intriguing approach to assess whether oxygen delivered to the tissues is effectively used by mitochondria. However, no routine methods are available to directly monitor cellular oxygen metabolism in clinical practice. Clinicians must therefore rely on monitoring methods that reflect cellular integrity and function, allowing indirect assessment (see surrogate measures below).

An experimental approach to assess cellular oxygen metabolism more directly is noninvasive measurement of mitochondrial oxygen partial pressure in superficial tissues by the protoporphyrin IX–triplet state lifetime technique.^74^ After topical application of 5-aminolevulinic acid, mitochondria accumulate protoporphyrin IX, and its delayed fluorescence lifetime inversely correlates with mitochondrial oxygen tension.^75^ This allows the estimation of mitochondrial oxygen partial pressure, providing direct insight into cellular oxygen metabolism.^76^^,^^77^ Another experimental technique involves broadband near-infrared spectroscopy to quantify the redox state of cytochrome c oxidase, the terminal enzyme in the mitochondrial electron transport chain.^78^ Changes in the oxidation state of this enzyme reflect shifts in mitochondrial oxygen availability and use.

### Surrogate measures for macrocirculation, microcirculation, and cellular oxygen metabolism

Several surrogate measures reflect overlapping aspects of the macrocirculation, microcirculation, and cellular oxygen metabolism and cannot be clearly assigned to a certain level of tissue oxygenation.

Processed EEG reflects neuronal electrical activity and can therefore provide indirect information about cerebral oxygenation and perfusion when disturbances are severe enough to affect neuronal function.^58^ A reduction in cerebral blood flow because of hypoperfusion can lead to characteristic changes in processed EEG, including a shift toward slower frequencies, burst suppression, or even complete suppression.^79^ Although these patterns are often associated with deep anaesthesia, their occurrence during episodes of haemodynamic instability can indicate cerebral hypoxia secondary to impaired cerebral perfusion.^80^ Processed EEG monitoring can therefore help detect clinically relevant cerebral hypoperfusion in real-time when interpreted alongside other variables.

Blood lactate concentration is the most widely used variable reflecting a mismatch between oxygen delivery and oxygen demand resulting from anaerobic metabolism.^81^ Serial lactate measurements can help monitor the adequacy of resuscitation and the response to therapeutic interventions.^81^^,^^82^ However, slow kinetics limit usefulness as a real-time marker of tissue hypoxia. In addition, elevated lactate concentrations are not specific for hypoxia, as they can also result from nonhypoxic mechanisms (e.g. beta-adrenergic stimulation, decrease in lactate clearance because of liver hypoperfusion or dysfunction, catecholamine-stimulated production, or alterations in pyruvate dehydrogenase activity).

Another surrogate measure of the circulation is central venous oxygen saturation, which reflects the balance between global oxygen delivery and consumption.^83^^,^^84^ It can be measured intermittently using blood samples obtained from a central venous catheter in the superior vena cava or continuously using specialised fibreoptic central venous catheters. Low central venous oxygen saturation (<65% to 70%) can indicate a mismatch between oxygen delivery and oxygen demand, whereas high central venous oxygen saturation can occur in the context of impaired oxygen extraction, such as in sepsis or mitochondrial dysfunction.^85^ Central venous oxygen saturation represents the venous oxygen saturation from the upper body and thus does not fully reflect global venous return or whole-body oxygen extraction, especially in conditions with significant circulatory redistribution. Mixed venous oxygen saturation, obtained from the pulmonary artery, mitigates some limitations of central venous oxygen saturation. Mixed venous oxygen saturation reflects the true mixed venous blood returning from the entire body, including the lower body, renal circulation, and coronary sinus; thus, it provides a more accurate estimate of the global balance between oxygen delivery and consumption. However, mixed venous oxygen saturation is not available in most surgical patients because pulmonary artery catheters are rarely used outside of cardiac surgery and liver transplantation.

Central venous-to-arterial carbon dioxide partial pressure difference is a further surrogate measure,^86^ which can provide information on carbon dioxide clearance at the tissue level. Under normal conditions, carbon dioxide is effectively cleared by blood flow. Consequently, an increased gap (>6 mm Hg) can reflect impaired tissue perfusion because of alterations in macrocirculation, microcirculation, or cellular oxygen metabolism.^87^ When interpreted alongside central venous oxygen saturation, the central venous-to-arterial carbon dioxide partial pressure gap can help differentiate between impaired oxygen delivery and impaired oxygen extraction. An increased gap combined with low central venous oxygen saturation suggests inadequate oxygen delivery because of impaired macrocirculation or microcirculation, whereas an increased gap with normal or high central venous oxygen saturation can suggest impaired oxygen extraction because of impaired cellular oxygen metabolism.

Tissue and intravascular microdialysis represent experimental techniques for assessing cellular metabolism and tissue oxygen use.^88^^,^^89^ Tissue microdialysis is an invasive method that continuously samples extracellular fluid, typically using probes placed in accessible tissues such as the brain, skeletal muscle, or subcutaneous adipose tissue, enabling real-time assessment of local tissue biochemistry,^90^ including measurement of extracellular metabolites such as lactate and pyruvate. Beyond interstitial probes, intravascular microdialysis systems integrated into central venous catheters enable continuous metabolic monitoring through central venous access.^89^ Microdialysis can help validate surrogate markers by linking alterations across macrocirculation, microcirculation, and cellular oxygen metabolism.

Several biochemical markers have been investigated as indicators of mitochondrial dysfunction or metabolic derangement. These include measurements of redox status (e.g. the ratio of nicotinamide adenine dinucleotide to its reduced form or the lactate-to-pyruvate ratio),^91^ inflammatory mediators (e.g. interleukin-6 and tumour necrosis factor alpha),^92^^,^^93^ and the ratio of reduced to oxidised glutathione.^94^ Although these approaches remain investigational, they represent important steps toward capturing a more comprehensive view of cellular oxygen metabolism. With further validation, they might complement established monitoring strategies and help ensure adequate tissue oxygenation in surgical patients.

Taken together, these surrogate measures offer practical tools for estimating the adequacy of tissue oxygenation, even though they cannot precisely localise the underlying cause of the oxygen imbalance. Interpretation requires careful integration with the clinical context, and combining multiple surrogate measures enhances diagnostic accuracy.

## Optimisation of tissue oxygenation

Although it is physiologically plausible that ensuring adequate tissue oxygenation is essential to prevent cellular dysfunction and organ failure, the most effective clinical strategies are uncertain. Potential targets for intraoperative therapeutic interventions exist across all levels of tissue oxygenation, including the macrocirculation, the microcirculation, and cellular oxygen metabolism.

### Macrocirculation

Clinicians most commonly target macrocirculatory variables during surgery. These interventions typically aim to increase oxygen delivery and perfusion pressure.

Cardiac output is a key determinant of oxygen delivery. Therefore, increasing cardiac output can improve oxygen delivery. Numerous small trials have suggested that perioperative cardiac output-guided therapy can mitigate organ injury and reduce postoperative complications, particularly infectious complications.^95^, ^96^, ^97^ However, in three recent multicentre trials,^98^, ^99^, ^100^ cardiac output-guided approaches did not reduce patient-centred postoperative complications. A major challenge with protocolised cardiac output-guided management is that there is no universal ‘normal’ value for cardiac output, as it is determined by metabolic demands, biometric characteristics, and comorbidities.^101^, ^102^, ^103^, ^104^ Consequently, what defines adequate cardiac output during surgery remains uncertain. Based on current evidence, cardiac output should not be routinely maximised but rather interpreted in the context of clinical and metabolic signs of tissue perfusion and oxygenation.^4^^,^^105^

Mean arterial pressure is the main determinant of organ perfusion pressure,^10^^,^^11^ and cohort studies have consistently shown that severe and prolonged intraoperative hypotension is associated with an increased risk for acute kidney injury and myocardial injury in high-risk patients.^106^, ^107^, ^108^, ^109^, ^110^ However, targeting intraoperative mean arterial pressure >60 to 65 mm Hg does not consistently reduce organ injury.^111^^,^^112^ Generally keeping intraoperative mean arterial pressure at higher levels (≥75 mm Hg or ≥80 mm Hg), compared to ≥60 to 65 mm Hg, did not reduce the incidence of adverse cardiovascular outcomes in major noncardiac surgery patients with high baseline risk for cardiovascular complications in a Swiss single-centre trial of 458 patients,^113^ an international multicentre trial of 7490 hypertensive patients,^114^ and a Chinese three-centre trial of 1500 patients.^115^ Similarly, keeping intraoperative mean arterial pressure >90 mm Hg, 80 mm Hg, or 70 mm Hg (depending on whether an individual patient’s risk for developing intraoperative hypotension was high, moderate, or low, respectively), compared to keeping mean arterial pressure ≥65 mm Hg, did not improve postoperative disability at 6 months after surgery or any secondary outcome in a 3522-patient trial (2306 included in the primary outcome analysis) performed in two Dutch centres.^116^

As baseline arterial pressure differs considerably among individuals presenting for surgery,^117^ it is plausible that individualising intraoperative arterial pressure targets based on patients’ preoperative baseline values could help reduce organ injury.^118^, ^119^, ^120^ However, in a German multicentre trial of 1142 major abdominal surgery patients,^121^ individualised perioperative arterial pressure management based on preoperative mean nighttime mean arterial pressure compared to routine blood pressure management with a mean arterial pressure intervention threshold of 65 mm Hg did not reduce the incidence of a composite primary outcome of acute kidney injury, acute myocardial injury, nonfatal cardiac arrest, or death within the first 7 days after surgery.

Further research should provide more evidence on whether targeting higher intraoperative mean arterial pressure^122^ or individualising mean arterial pressure based on cerebral blood flow autoregulation data^123^ can help prevent organ injury. Based on the available observational studies and trials, it is currently reasonable to recommend keeping intraoperative mean arterial pressure >60 mm Hg,^4^^,^^47^ the population harm threshold for acute kidney injury and myocardial injury.^106^, ^107^, ^108^^,^^124^^,^^125^

Defining appropriate intraoperative targets is only one part of arterial pressure management; it is just as important to select the right intervention to maintain adequate mean arterial pressure. Intraoperative hypotension is not a disease entity but rather a clinical sign indicating profound haemodynamic disturbance. Intraoperative hypotension has various underlying causes, including vasodilation, hypovolaemia, bradycardia, and myocardial depression that result in different ‘endotypes of hypotension’.^126^^,^^127^ Perioperatively, these hypotension endotypes are generally distinguishable, although they can overlap and change over time.^126^^,^^127^ In clinical practice, identifying these ‘endotypes of hypotension’^126^^,^^127^ is important because optimal treatment strategies should be tailored to the specific underlying haemodynamic alterations.^4^^,^^47^ *Vasodilation* often requires vasopressors such as norepinephrine to restore vascular tone and maintain perfusion pressure. However, vasopressors should be used judiciously, as high doses can impair microcirculatory flow and increase the risk of acute kidney injury.^128^, ^129^, ^130^, ^131^ In patients with *hypovolaemia*, fluid administration can help restore cardiac output and mean arterial pressure. Nevertheless, excessive fluids can lead to tissue oedema and impaired oxygen diffusion.^132^ Although there is no generally accepted definition of intraoperative *bradycardia*,^133^ low heart rate resulting in profound hypotension or low cardiac output can necessitate chronotropic support.^4^ *Myocardial depression*, which in surgical patients can be caused or aggravated by anaesthetic drugs, can be treated with inotropes in selected patients.

Besides cardiac output, arterial oxygen content is a key determinant of oxygen delivery. Red blood cell transfusion increases haemoglobin concentration, thereby increasing arterial oxygen capacity. However, the impact of blood transfusion on oxygen delivery is often modest, as the associated rise in blood viscosity can increase vascular resistance and reduce cardiac output, offsetting the benefit of increased arterial oxygen content.^134^ As a result, transfusion rarely leads to a meaningful improvement in tissue oxygenation.^135^ Blood transfusion should thus be reserved for carefully selected indications, given potential risks of transfusion-transmitted infections, immunological reactions, transfusion-associated circulatory overload, and transfusion-related acute lung injury.^136^, ^137^, ^138^ Optimal haemoglobin thresholds for perioperative blood transfusion remain a matter of ongoing research.^139^ In addition, it remains to be assessed whether combining haemoglobin thresholds with other variables, such as central venous oxygen saturation, or near-infrared spectroscopy-derived tissue oxygen saturation can help to guide transfusion decisions.^140^, ^141^, ^142^

In anaemic surgical patients, erythropoiesis-stimulating agents such as erythropoietin can be administered before surgery to increase haemoglobin concentrations and thereby the oxygen-carrying capacity of blood.^143^, ^144^, ^145^ However, evidence for effectiveness in the perioperative setting remains limited, and concerns such as thrombotic risk must be considered.^146^

### Microcirculation

Interventions to improve the macrocirculation should be complemented by strategies targeting the microcirculation and cellular oxygen metabolism. Commonly used macrocirculatory interventions may also influence microcirculatory perfusion, but their effects on the microcirculation are variable and often poorly understood.

Judicious fluid therapy can enhance mean arterial pressure and cardiac output and microcirculatory perfusion, provided that interstitial oedema is avoided.^16^ Conversely, fluid overload or excessive haemodilution can impair oxygen diffusion and microvascular flow. Similarly, vasoactive agents can restore perfusion pressure but at high doses can induce microvascular vasoconstriction and microvascular flow heterogeneity.^16^^,^^147^ These examples highlight the need for a physiology-guided, cause-specific approach that takes both macrocirculatory and microcirculatory consequences into account.

Whether therapies can or should specifically target the microcirculation in surgical patients remains uncertain. Most research on microcirculatory-directed interventions has been conducted in critically ill patients in intensive care settings. In this context, microcirculatory alterations are common and correlate with poor outcomes, particularly in patients with systemic inflammation or prolonged shock.^148^ However, even in critically ill patients, the effect of interventions specifically targeting microcirculatory perfusion remains poorly understood.^149^ In the largest trial to date, haemodynamic management guided by sublingual microcirculation monitoring did not reduce 30-day mortality.^150^

In contrast, microcirculatory function appears to be preserved during elective surgery. Observational studies have shown that, unlike in critical illness, the (sublingual) microcirculation is not impaired in patients presenting for elective surgery^151^^,^^152^ and that it is preserved and remains largely functional during noncardiac surgery with general anaesthesia.^151^, ^152^, ^153^, ^154^ Only a few randomised trials have investigated whether perioperative haemodynamic strategies affect microcirculatory flow. In a trial including 31 patients having major abdominal surgery, the sublingual microcirculation did not substantially differ between patients assigned to pulse pressure variation/cardiac index-guided *vs* mean arterial pressure-guided haemodynamic management.^155^ This is in line with another trial in 67 patients having major abdominal surgery revealing that there were no important differences in sublingual microvascular flow index among patients randomised to maximising stroke volume, maintaining a preoperative resting cardiac index, or routine care during and for the first 6 h after surgery.^156^ In contrast, in a randomised trial in 86 patients having high-risk abdominal surgery, an assisted fluid management strategy improved sublingual microvascular flow compared with a routine fluid management strategy.^157^ The assisted fluid management strategy was associated with a higher microvascular flow index, improved cardiac output, and lower lactate concentrations, suggesting that an adequate fluid management strategy can help preserve microcirculatory perfusion.^157^ The effects of blood transfusion on microcirculatory perfusion remain incompletely understood. Dysfunctional interactions between stored erythrocytes and the microvasculature can even result in impaired microcirculatory perfusion following blood transfusion.^158^

### Cellular oxygen metabolism

Ensuring adequate cellular oxygen metabolism represents the final critical step in ensuring sufficient tissue oxygenation. This requires intact mitochondrial function and the availability of metabolic substrates, as well as the absence of conditions that impair oxidative phosphorylation, such as inflammation.

Therapeutic strategies aiming to directly improve oxygen use at the cellular level are evolving. Mitochondrial function can be supported indirectly by ensuring normoglycaemia, avoiding excessive oxygen exposure (which can increase reactive oxygen species), and maintaining redox balance.^159^

Erythrocyte transfusion is expected to improve tissue oxygenation and mitochondrial function; however, supporting evidence remains limited. In an observational study, transfusion did not increase mitochondrial oxygen tension in 63 critically ill patients.^160^ However, erythrocytes contribute not only to oxygen transport but also to the regulation of oxidative stress and mitochondrial function through glutathione recycling and inflammatory modulation.^161^ Impaired erythrocyte function might therefore negatively impact cellular oxygen metabolism.

Intravenous iron can increase haemoglobin concentrations and improve oxygen-carrying capacity in patients with iron-deficiency anaemia.^162^ Beyond this systemic effect, intravenous iron might also support mitochondrial respiration and improve cellular oxygen use, suggesting that some interventions benefit both systemic delivery and cellular use of oxygen.^163^ This might explain why iron improves symptoms and physical performance in heart failure patients with iron deficiency, even in the absence of anaemia.^164^

Although promising experimental techniques such as noninvasive measurement of mitochondrial oxygen partial pressure and broadband near-infrared spectroscopy offer direct insights into mitochondrial oxygenation, they are not yet suitable for routine clinical use. Nonetheless, understanding the determinants of oxygen consumption at the cellular level will be essential to close the loop of tissue oxygenation optimisation.

## Conclusions

Adequate tissue oxygenation is essential for maintaining organ function during and after surgery. While most clinical strategies focus on the macrocirculation, tissue oxygenation also depends on the microcirculation and cellular oxygen metabolism. Monitoring and treatment should therefore address the complete pathway of tissue oxygenation. Future research is needed to better understand how to target and support each level to improve outcomes in surgical patients.

## Authors’ contributions

Literature review: all authors.

Drafting the manuscript: all authors.

Critical revision of the manuscript for important intellectual content: all authors.

Final approval of the version to be published: all authors.

Agreement to be accountable for all aspects of the work thereby ensuring that questions related to the accuracy or integrity of any part of the work are appropriately investigated and resolved: all authors.

## Funding

The idea for this project and manuscript was developed during a hybrid (in-person and virtual) meeting in Brussels, Belgium, on November 6, 2024, organised by the Network for the Advancement of Patient Blood Management, Haemostasis and Thrombosis (NATA). The project was supported by an unrestricted grant from Masimo (Irvine, CA, USA). Masimo was not involved in the literature review, the writing of the manuscript, or the decision to submit the manuscript for publication.

## Declaration of generative AI and AI-assisted technologies in the writing process

During the preparation of this work, the authors used ChatGPT GPT-4.1 (OpenAI, San Francisco, CA, USA) to improve readability and language. The authors reviewed and edited the content as needed and take full responsibility for the content of the publication.

## Declaration of interests

JM is a member of the Board of Directors of the Network for the Advancement of Patient Blood Management, Haemostasis and Thrombosis (NATA). SL has received honoraria for giving lectures from Pharmacosmos (Holbæk, Denmark), CSL Vifor (Villars-sur-Glâne, Switzerland), Masimo (Neuchâtel, Switzerland), and Pfizer (Paris, France), and has received nonfinancial research support from Pharmacosmos, and is a member of the Board of Directors of the Network for the Advancement of Patient Blood Management, Haemostasis and Thrombosis (NATA). PM has received honoraria for giving lectures from Biotest AG (Dreieich, Germany), CSL Behring GmbH (Hattersheim, Germany), Pharmacosmos GmbH (Wiesbaden, Germany), CSL Vifor GmbH (Munich, Germany), and is a member of the Board of Directors of the Foundation for Health, Patient Safety and Patient Blood Management (PBM Foundation), a member of the Board of Directors of the Network for the Advancement of Patient Blood Management, Haemostasis and Thrombosis (NATA) and a member of the Working Group of the Scientific Advisory Board “Cross-sectional Guidelines for Therapy with Blood Components and Plasma Derivatives”. DF has received honoraria for giving lectures from CSL Behring (King of Prussia, PA, USA; speaker fees paid to WFSA), and an unrestricted grant from CSL Vifor (London, UK) to the WFSA. TH is an advisory board member for CSL Behring (King of Prussia, PA, USA), Octapharma (Lachen, Switzerland), CERUS (Concord, CA, USA), and Grifols (Barcelona, Spain), and is a member of the Board of Directors of the Network for the Advancement of Patient Blood Management, Haemostasis and Thrombosis (NATA). JP has received honoraria for giving lectures from Edwards Lifesciences (Nyon, Switzerland) and Masimo (Irvine, CA, USA), serves as an advisor for RDS (Strasbourg, France), and is a member of the Network for the Advancement of Patient Blood Management, Haemostasis and Thrombosis (NATA). ER has received personal fees and nonfinancial support for conference participation from CSL Vifor (Paris La Défense, France), Masimo (Neuchâtel, Switzerland), and Pfizer (Paris, France), has received honoraria from the French National Authority for Health (HAS) for his role as project manager of the 2022 French guidelines on perioperative blood management, and is a member of the Scientific Committee of the Network for the Advancement of Patient Blood Management, Haemostasis and Thrombosis (NATA). SR has received honoraria for lectures and support for congress travel and accommodation from Masimo (Irvine, CA, USA), Vygon (Écouen, France), Viatris (Milan, Italy), B. Braun (Melsungen, Germany), Baxter (Deerfield, IL, USA), Medtronic (Minneapolis, MN, USA), and Kures (Milan, Italy), and is a member of the Advisory Boards of Kures (Milan, Italy) and Viatris (Milan, Italy). AB has no conflict of interest to declare. BS is a consultant for Edwards Lifesciences (Irvine, CA, USA), Philips North America (Cambridge, MA, USA), GE Healthcare (Chicago, IL, USA), Vygon (Aachen, Germany), Masimo (Neuchâtel, Switzerland), Retia Medical (Valhalla, NY, USA), Maquet Critical Care (Solna, Sweden), Pulsion Medical Systems (Feldkirchen, Germany), Dynocardia (Cambridge, MA, USA), and RDS (Strasbourg, France), has received restricted research grants from Edwards Lifesciences, Baxter (Deerfield, IL, USA), GE Healthcare, Masimo, Philips Medizin Systeme Böblingen (Böblingen, Germany), CNSystems Medizintechnik (Graz, Austria), Pulsion Medical Systems, Vygon, Retia Medical, Osypka Medical (Berlin, Germany), has received honoraria for giving lectures from Edwards Lifesciences, Philips Medizin Systeme Böblingen, Baxter, GE Healthcare, Masimo, CNSystems Medizintechnik, Getinge (Gothenburg, Sweden), Pulsion Medical Systems, Vygon, Ratiopharm (Ulm, Germany), and is an editor of the *British Journal of Anaesthesia*.
